# Morphofunctional parameters, physical fitness and musculoskeletal symptoms in cooperative recyclers

**DOI:** 10.47626/1679-4435-2023-827

**Published:** 2023-04-18

**Authors:** Raiane Caroline Garcia, Natália Quevedo dos Santos, Daniela Eloísa do Nascimento, Mateus Dias Antunes, Vera Lúcia Kerber, Sônia Maria Marques Gomes Bertolini

**Affiliations:** 1 Departamento de Pós-Graduação Strictu Sensu em Promoção da Saúde, Universidade Cesumar, Maringá, PR, Brazil; 2 Curso de Fisioterapia, Universidade Cesumar, Maringá, PR, Brazil; 3 Departamento de Fisioterapia, Fonoaudiologia e Terapia Ocupacional, Faculdade de Medicina, Universidade de São Paulo, São Paulo, SP, Brazil; 4 Instituto Cesumar de Ciência, Tecnologia e Inovação ICETI, Universidade Cesumar, Maringá, PR, Brazil

**Keywords:** health status, solid waste, occupational health, health promotion, condições de saúde, resíduos sólidos, saúde do trabalhador, promoção da saúde

## Abstract

**Introduction:**

Members of solid waste recycling cooperatives are exposed to serious conditions and complications in their everyday life, which makes them likely to present poor quality of life and unfavorable health conditions in their work environment.

**Objectives:**

To evaluate morphofunctional parameters, physical fitness, and musculoskeletal symptoms of workers at solid waste recycling cooperatives in Maringá, state of Paraná, Brazil.

**Methods:**

This was a quantitative, cross-sectional, descriptive study. Data were collected from 60 cooperative members of both sexes linked to the Popular and Solidarity Recycling Association of Maringá. Participants underwent a medical screening at the cooperative, involving anamnesis, pulmonary and cardiac auscultation, and blood pressure measurement. In a second moment, they underwent physical assessment in the laboratory, using instruments for physical tests and questionnaires.

**Results:**

There was a predominance of females in the sample (54%), with a mean age of 41.82±12.03 years, and most participants did not practice physical activity (70%). With regard to body composition, women had the highest body mass index (28.29±6.61 kg/m^2^); as for the variables physical and aerobic fitness, men showed better scores than women (p < 0.05). In relation to musculoskeletal symptoms, most participants complained of lower back pain (56.66%).

**Conclusions:**

Although the results for anthropometric variables are within normal standards in most cooperative members, most of them present with musculoskeletal symptoms and do not practice physical activity, which can have negative implications in their health conditions in the medium and long term.

## Abstract (pt)

**Introdução:**

Os cooperados de reciclagem de resíduos sólidos são expostos a gravidades e complicações no seu dia a dia. Diante disso, é possível que apresentem uma má qualidade de vida e condições de saúde não favoráveis em seu âmbito de trabalho.

**Objetivos:**

Avaliar os parâmetros morfofuncionais, a aptidão física e os sintomas osteomusculares dos trabalhadores das cooperativas de reciclagem de resíduos sólidos de Maringá, no estado do Paraná.

**Métodos:**

Tratou-se de estudo quantitativo do tipo transversal descritivo. Foram coletados dados de 60 cooperados, de ambos os sexos, vinculados à Associação de Reciclagem Popular e Solidária de Maringá. Os participantes foram submetidos a uma triagem médica na cooperativa, envolvendo anamnese, ausculta pulmonar e cardíaca e mensuração da pressão arterial. Em um segundo momento, foram submetidos à avaliação física em laboratório, com a utilização de instrumentos para testes físicos e preenchimento de questionários.

**Resultados:**

Na amostra, houve predomínio do sexo feminino (54%), com média de idade de 41,82±12,03, e a maioria não praticava exercícios físicos (70%). Quanto à composição corporal, o sexo feminino apresentou maior índice de massa corporal (28,29±6,61 kg/m^2^); para as variáveis de aptidão física e aeróbia, o sexo masculino apresentou melhores escores em relação ao sexo feminino (p < 0,05). Em relação aos sintomas osteomusculares, a maioria relatou sintomas de dor na parte inferior das costas (56,66%).

**Conclusões:**

Apesar de os resultados das variáveis antropométricas da maioria dos cooperados encontrarem-se dentro dos padrões de normalidade, a maioria apresenta sintomas osteomusculares e não pratica atividades físicas, o que pode ter implicações negativas nas suas condições de saúde a médio e longo prazo.

## INTRODUCTION

Capitalism encourages society to be increasingly more consumerist, which implies major environmental impacts, such as increased generation of solid waste. However, in general, this system neglects the recovery and reuse of this waste. In this scenario, public authorities have transferred the task of managing waste to vulnerable workers (waste collectors), excluded from the formal labor market.^1-3^ These workers eventually assume the role of environmental agents of great importance to recycling, driven by the need for survival, which leaves them with practically no freedom to make their own choices and make use of their capabilities with autonomy.^4^

Recycling cooperatives are institutions that work in the management, separation, and treatment of solid waste in order to provide it with a new commercial purpose, thus reinserting it in the chain of goods production.^5^ These cooperatives have a different hiring system, compared with the traditional hiring process adopted in privatized services; furthermore, they favor social insertion of a portion of workers with few resources, who often do not find a position in the formal labor market.^5,6^

In this sense, this working class eventually experiences situations of insecurity, leading to economic restrictions and even poverty.^3^ Therefore, waste collectors become vulnerable, which poses health risk factors, because they are constantly exposed to inappropriate environments. Also as a consequence of their work, contaminations and damages to their physical integrity may occur, due to some accidents when handling wastes; moreover, they are likely to become carriers and transmitters of diseases.^7,8^

The World Health Organization (WHO) defines health as a “state of complete physical, mental and social wellbeing and not merely the absence of disease or infirmity.”^9^ Health promotion is presented as a set of strategies to improve population’s quality of life and health conditions.^10^ In this context, the National Health Promotion Policy (Política Nacional de Promoção da Saúde, PNPS), established in 2015, aims to promote equity and better life conditions for individuals and populations, working on social health determinants, which include sustainability and care with the environment. Thus, it can be observed that the PNPS is concerned with the environmental issue and with sustainability as factors that influence on individuals’ wellbeing. However, organization managers did not often assess life conditions in the workplace, thus being unable to diagnose possible problems so as to propose measures to improve determinants of population health.

In light of the foregoing, it is pertinent to conduct studies to identify the actual situation of the aforementioned population, in order to implement future actions to improve health conditions. Therefore, the aim of the present study was to assess morphofunctional parameters, physical fitness, and musculoskeletal symptoms of workers at solid waste recycling cooperatives in the city of Maringá, state of Paraná, Brazil.

## METHODS

This was a quantitative, observational, cross-sectional, descriptive study complying with Strengthening the reporting of observational studies in epidemiology (STROBE)^11^ guidelines and conducted from October to December 2019, with a sample of members of Cooper Norte and Cooper Palmeiras cooperatives, both registered in Popular and Solidarity Recycling Association of Maringá (ARPSOL). Sixty cooperative members participated (32 female and 28 male), aged 18 years or above and who were available to perform all physical tests. Exclusion criteria were performing only administrative activities or be absent during the data collection period.

Data collection took place at two moments. In the first one, a medical screening was conducted at the cooperatives, involving the application of a sociodemographic questionnaire, anamnesis, pulmonary and cardiac auscultation, and blood measurement, all procedures performed according to the standards established in 7^th^ Brazilian Guidelines on Arterial Hypertension.^12^ In a second moment, physical tests were conducted at Laboratório Interdisciplinar de Intervenção em Promoção da Saúde (LIIPS) of Universidade Cesumar, in order to gather data related to: (1) anthropometry and body composition; (2) maximal isometric handgrip strength; (3) maximal isometric lumbar traction strength; (4) maximal isometric lower limb muscle strength; (5) flexibility; (6) aerobic fitness; and (7) self-reported musculoskeletal symptoms.

Anthropometry and body composition were assessed by the InBody 570^®^ eight-electrode tetrapolar bioimpedance analyzer, in order to obtain the following parameters: a) body weight; b) waist-hip ratio; c) total body water; d) body fat mass; e) musculoskeletal mass; f) basal metabolic rate (BMR); and g) body mass index (BMI).

Maximal handgrip strength was measured using a handgrip dynamometer. Maximum isometric lumbar and lower limb muscle strengths were measured using a hydraulic dynamometer. Posterior chain flexibility was assessed by the sit and reach test on a Wells bench. Aerobic fitness was evaluated by the maximum oxygen volume (VO_2max_) obtained in the modified Bruce protocol on a treadmill, with 1-minute stages until voluntary exhaustion.

Musculoskeletal symptoms were assessed using the Nordic Musculoskeletal Questionnaire, translated and adapted to Portuguese by Barros & Alexandre (2003), which contains the diagram of a human figure from a posterior view, divided into nine anatomical regions. Participants answer whether they felt pain, tingling, or numbness, were not able to perform normal activities, or had an appointment with a health care professional in the last 12 months and in the last 7 days; moreover, participants were also asked whether they had some problem in the nine anatomical regions during the last 7 days.

Data analysis was initially performed by storing data in Excel software, version 2016. Data normality was first tested by the Shapiro-Wilk test, and subsequently by the Kolmogorov-Smirnov test. After normality was confirmed, descriptive statistics involved calculating mean, standard deviation, and absolute and relative frequencies of all variables, in addition to the Student’s *t* test to compare numerical variables in relation to sex. All analyses were made using the SPSS 26.0^®^ statistical package . The level of significance was set at 5%.

This study was approved by Ethics Committee, under opinion number 2.965.086/20. Furthermore, cooperative members received information on all research procedures, all questions were clarified, and then participants signed the informed consent form.

## RESULTS

Sixty cooperative members participated in the study, aged from 18 to 65 years, with a mean age of 40.25±13.92 years and no significant difference between the sexes (p = 0.46). Table 1 shows the distribution of sociodemographic and morphofunctional variables of cooperative members, highlighting the high percentage of participants who did not practice physical activity (70%).

**Table 1:** Distribution of participants according to sociodemographic characteristics and morphofunctional variables (n = 60)

Variables	Frequency (n)	Percentage (%)
Sex		
Male	28	46.00
Female	32	54.00
Age (years)		
18 to 29	18	30.00
30 to 39	9	15.00
40 to 49	16	26.67
50 to 59	12	20.00
60 or older	5	8.33
Schooling		
Incomplete primary education	24	41,00
Complete primary education	15	25.00
Complete high school education	9	14.30
Incomplete higher education	9	14.30
Complete higher education	3	5.40
Monthly income (Brazilian reais)		
Up to 954.00	21	36.40
From 955.00 to 2,862.00	36	60,00
2,863.00 or more	3	3.60
Marital status		
Single	23	36.90
Married	25	42.60
Divorced	7	11.50
Widower	5	9,00
Glucose levels		
Normal	42	70,00
Pre-diabetes	15	25,00
Diabetes	3	5,00
Smoking		
Yes	25	41.70
No	35	53.30
Alcohol consumption		
Yes	29	48,00
No	31	52,00
Systolic blood pressure		
Normal	36	60,00
Pre-hypertension	19	31.66
Hypertension	5	8.34
Diastolic blood pressure		
Normal	42	70,00
Pre-hypertension	14	23.33
Hypertension	4	6.66
Pulmonary auscultation		
With changes	6	10,00
Without changes	54	90,00
Cardiac auscultation		
With changes	0	0
Without changes	60	100,00
Practices physical activities		
Yes	18	30,00
No	42	70,00

Table 2 presents variables related to anthropometric measures, body composition, physical fitness, and aerobic fitness stratified by sex, showing higher rates of overweight in women and higher physical fitness rates in men (p < 0.005).

**Table 2:** Anthropometric measures, body composition, physical fitness, and aerobic fitness stratified by sex (n = 60)

Variable	Men (46.7%)	Women (53.3%)	t test p-value
	Mean ± SD	Mean ± SD	
Anthropometric measures			
Age	35.54 ± 13.40	41.82 ± 12.03	0.46
Height (m)	172.33 ± 7.71	159.32 ± 4.79	0.00^*^
Body composition			
Body weight (kg)	73.73 ± 11.61	71.54 ± 15.73	0.88
BMI (kg/m^2^)	24.86 ± 3.79	28.29 ± 6.61	0.03^*^
Waist-hip ratio (cm)	0.87 ± 0.10	0.82 ± 0.09	0.01^*^
Total body water (L)	39.37 ± 5.46	32.85 ± 5.49	0.00^*^
Body fat mass (kg)	18.18 ± 9.09	28.49 ± 10.56	0.00^*^
Musculoskeletal mass (kg)	29.91 ± 4.62	24.00 ± 3.02	0.00^*^
BMR (kcal)	1,525.84 ± 162.92	1,315.44 ± 108.31	0.00^*^
Physical fitness			
MIHS-DH (kgf)	43.20 ± 7.57	24.40 ± 5.07	0.00^*^
MIHS -NDH (kgf)	41.58 ± 7.09	23.55 ± 6.51	0.00^*^
MILTS (kgf)	123.13 ± 21.56	71.18 ± 20.20	0.00^*^
MILLMS (kgf)	118.17 ± 22.48	64.94 ± 16.83	0.00^*^
Sit and reach (reps)	20.33 ± 4.73	18.47 ± 5.01	0.01^*^
Flexibility (cm)	29.65 ± 8.06	24.03 ± 9.10	0.15
Aerobic fitness			
VO^2^max (mL/kg/min)	59.00 ± 16.00	46.00 ± 10.00	0.00^*^

BMI = body mass index; BMR = basal metabolic rate; SD = standard deviation; MIHS-DH = maximal isometric handgrip strength of the dominant hand; MIHS-NDH = maximal isometric handgrip strength of the nondominant hand; MILLMS = maximal isometric lower limb muscle strength; MILTS = maximal isometric lumbar traction strength; VO_2max_= maximum oxygen volume.

* Significant differences were observed for the variables analyzed (p < 0.05).

Table 3 describes the musculoskeletal symptoms reported in the last 12 months and in the last 7 days. There was a higher occurrence of lower back pain (56.66%).

**Table 3:** Distribution of participants according to presence of pain in different body segments (n = 60)

Body segments	Pain in the last 12 months			Pain in the last 7 days		
	Total n (%)	Men n (%)	Women n (%)	Total n (%)	Men n (%)	Women n (%)
Neck	20 (33.33)	7 (22.58)	13 (40.63)	15 (25.00)	6 (19.35)	9 (28.13)
Shoulders	28 (46.66)	13 (41.94)	15 (46.88)	20 (33.33)	8 (25.81)	11 (34.38)
Upper back	23 (38.33)	7 (22.58)	16 (50.00)	14 (23.33)	5 (16.13)	9 (28.13)
Elbows	13 (21.66)	6 (19.35)	7 (21.88)	11 (18.33)	6 (19.35)	5 (15.63)
Wrists/hands	27 (45.00)	12 (38.71)	15 (46.88)	18 (30.00)	9 (29.03)	8 (25.00)
Lower back	34 (56.66)	19 (61.29)	17 (53.13)	24 (40.00)	12 (38.71)	12 (37.50)
Hip/thighs	19 (31.66)	7 (22.58)	11 (34.38)	16 (26.66)	5 (16.13)	10 (31.25)
Knees	22 (36.66)	11 (35.48)	11 (34.38)	15 (25.00)	8 (25.81)	7 (21.28)
Ankles/feet	23 (38.33)	11 (35.48)	14 (43.75)	17 (28.33)	9 (29.03)	10 (31.25)

## DISCUSSION

The main results of this study show that members of the solid waste recycling cooperative had normal values in morphofunctional variables. In the literature consulted, no studies were found assessing these variables, although our results may be justified by the fact that the sample consisted of a young adult population.

In the present study, most cooperative members were female and married, consistent with the studies by Lutinski et al.^13^ and Bonini-Rocha et al.^14^ In this sense, this professional activity is considered as a strategy of social inclusion for this part of the population, who lives in vulnerable conditions.^13^ With regard to schooling, a study by Conceição & Silva^15^ showed that low schooling increases the number of informal workers, leading to the hypothesis that they are recyclable waste pickers. In agreement with the present study, several authors report low schooling levels in workers and recyclable waste pickers.^16-18^ Investigations conducted by the Brazilian National Association of Recyclable Waste Pickers (Associação Nacional dos Catadores e Catadoras de Materiais Recicláveis, ANCAT) reveal that, in Brazil, 60% of cooperative members had completed primary education.^19^

With regard to health related issues, research participants had glucose and blood pressure levels within normal standards; however, changes is these levels are known to represent a growing health problem for the world community and have a multifactorial etiology; furthermore, high glucose levels may be considered the third leading cause of early death.^20^ It is also estimated that uneducated individuals or with incomplete primary education and low income show a higher prevalence of hypertension and diabetes mellitus.^20^ In terms of body composition, BMI is an anthropometric index related to nutritional status and widely used at the population level.^21^ Therefore, obesity is closely linked to high BMI values, increasing the prevalence of different non-communicable chronic diseases, impairing health and quality of life.^22^ In the current study, overweight is more prominent in women.

In a research that evaluated physical fitness levels among garbage collectors in the city of Vitória, state of Espírito Santo, Brazil, Araújo^23^ assessed dorsal muscle and lower limb strength in 17 individuals and found a mean of 143 kgf (±21.85), a value different from those observed in the present study. This discrepancy in values may be related to the fact that this study had a significantly larger sample size, despite using a similar methodology to carry out the test. Aerobic fitness depends on cardiovascular function, but also reflects the role of lower limb strength to perform walking.^24^ According to the literature, the present study showed differences in aerobic fitness between the sexes and are within normal standards; moreover, results found that VO_2max_ decreases with age_._^25^ It was observed that 70% of study participants did not practice physical exercises, although the literature shows that regular physical activity brings physical and mental benefits to the human body, improving pain and quality of life.^26^

With regard to work-related musculoskeletal symptoms, all cooperative members reported symptoms in the last 12 months in at least one of the nine body regions, which is consistent with studies conducted in different countries.^27,28^ This study observed a higher prevalence of symptoms in the lower back (lumbar region), a result similar to that of the studies by Bonini-Rocha et al.^14^ and Araújo & Sato,^29^ conducted in São Paulo, Brazil, with recyclable waste pickers, showing the predominance of lower back pain, followed by pain in the shoulders, neck, ankles, and wrists/hands. These are highly demanded body regions, due to the need of these workers to remain in the same position for a long time, to move heavy loads, and to constantly mobilize their upper limbs.^28^ These data corroborate a study that investigated a population of municipal solid waste collectors and found a correlation between stress and pain, in which those who reported moderate to intense pain had higher levels of stress compared to those with mild pain.^27^ Of note, there was a high presence of lower back pain in several studies addressing this population.^14,29^

It is worth highlighting that, after data collection was concluded, cooperative members whose results were outside normal standards were immediately referred for a medical appointment or treated and monitored by the family health team. Furthermore, study limitations are particularly related to the fact that it is not possible to determine causative relationships between study variables and the work performed by waste pickers, due to the cross-sectional design of the study, in which cause and effect are observed at the same time. The results suggest the need of further qualitative studies with informal cooperative members, in order to better understand occupational risks, perceptions about work, and difficulties so as to reinforce the importance of waste pickers’ work to solid waste policy makers and to the society.

However, it is important to emphasize that, based on the data obtained, it will be possible to take a more detailed look on the needs and difficulties inherent to this working class, contemplating not only its occupational activity, but encompassing a biopsychosocial care model, thus providing opportunities to implement measures aimed at establishing practices related to ergonomic education, occupational safety, improvements in the workplace, and greater care and understanding about deep-rooted social and psychosocial issues that provide support to diverse chronic damages to the health of this population. Therefore, a possibility of engagement is offered by means of studies and measures to support cooperative members, in addition to providing health professionals with deeper understanding about the great deprivation experienced by this population, thus improving approach, guidance, and care to patients.

## CONCLUSIONS

It was concluded that the members of a solid waste cooperative have good health conditions, i.e., above the expected levels. However, despite favorable results regarding health conditions, most cooperative members had musculoskeletal symptoms and did not practice physical activities, which can have negative implications for their health conditions in the medium and long term. Knowledge of this risk factor that might contribute to the development of prevention and intervention programs directed to pain in body segments affected by work activities in this population. In this sense, it bears emphasizing the importance of educational guidance to provide benefits to the general state of health, especially physical, thus reducing musculoskeletal symptoms.
